# A histogram of [^18^F]BBPA PET imaging differentiates non-neoplastic lesions from malignant brain tumors

**DOI:** 10.1186/s13550-024-01069-7

**Published:** 2024-02-02

**Authors:** Ziren Kong, Zhu Li, Junyi Chen, Yixin Shi, Nan Li, Wenbin Ma, Yu Wang, Zhi Yang, Zhibo Liu

**Affiliations:** 1 Department of Neurosurgery, Peking Union Medical College Hospital, Chinese Academy of Medical Sciences and Peking Union Medical College, Beijing, China; 2 Department of Head and Neck Surgery, National Cancer Center/National Clinical Research Center for Cancer/Cancer Hospital, Chinese Academy of Medical Sciences and Peking Union Medical College, Beijing, China; 3 Key Laboratory of Carcinogenesis and Translational Research, Department of Nuclear Medicine, Peking University Cancer Hospital and Institute, Beijing, China; 4 National Laboratory for Molecular Sciences, Radiochemistry and Radiation Chemistry Key Laboratory of Fundamental Science, NMPA Key Laboratory for Research and Evaluation of Radiopharmaceuticals, Key Laboratory of Bioorganic Chemistry and Molecular Engineering of Ministry of Education, College of Chemistry and Molecular Engineering, Peking University, BeijingBeijing, China; 5 Peking University-Tsinghua University Center for Life Sciences, Beijing, China; 6 Changping Laboratory, Beijing, China

## Introduction

Differentiating treatment response from tumor progression is fundamental but challenging for almost all oncological subjects, as the treatment strategy is effective and should be insisted in the former situation, while the therapeutic regimen is invalid and necessitates substitutions in the latter circumstances [1]. However, radiotherapy or immunotherapy may induce pseudo-progression, a transient increase of tumor volume due to tumor cell lysis or immune cell infiltration followed by delayed tumor shrinkage, and is difficult for early clinical and radiological identification [1, 2]. In malignant brain tumors, 10–30% of tumors showed pseudo-progression following radiotherapy, immunotherapy and targeted therapy [3–6], some of which were not restricted to the recent onset of treatment [7, 8]. In addition, alternative non-neoplastic conditions such as radiation necrosis or inflammation may also mimic neoplasms and warrant appropriate distinction [3]. Response Evaluation Criteria in Solid Tumors (RECIST) and Response Assessment in Neuro­Oncology (RANO) have been proposed [9, 10], yet the performance in distinguishing treatment response from tumor progression remains to be improved [11–14].

Boramino acids (BAA) are a class of amino acid biosimilars with the boron trifluoride group (–BF_3_) to replace the carboxyl group (–COOH) of amino acids, which mimics the corresponding amino acid in biological recognition and transportation [15]. The ^18^F-^19^F isotope exchange reaction of boron trifluoride moiety allows the molecule to be mildly radiolabeled and can facilitate tumor theranostics through identical chemical structure (the only difference between positron emission tomography [PET] diagnosis and boron neutron capture therapy [BNCT] for treatment is ^18^F or ^19^F) [15–20]. The first-in-human study of this class of PET tracers demonstrated sufficient safety, clean background and high tumor activity in malignant brain tumors [21, 22], validating the concept and potential clinical value of boron amino acids. Subsequently, trifluoroborate boronophenylalanine (BBPA) that replaced the carboxyl group (–COOH) of 4‑boronophenylalanine (BPA) with boron trifluoride group (-BF_3_) was synthesized and is recognized as the next generation of boron amino acids thanks to the doubled boron delivery efficiency [23].

This study raised a [^18^F]BBPA PET-based approach to differentiate non-neoplastic lesions from proliferating tumors, aiming to provide a non-invasive method to uncover true lesion property. A total of 21 patients were included and underwent [^18^F]BBPA PET and contrast-enhanced magnetic resonance imaging (MRI) scans. Both neoplastic and non-neoplastic lesions exhibited elevated [^18^F]BBPA radioactivity and cannot be distinguished by traditional parameters. Histograms of the standard uptake value (SUV) within region of interest (ROI) were plotted, and the malignant tumors exhibited a symmetrical distribution (similar to normal distribution), while the non-neoplastic lesions displayed a positive skewed (left deviated) distribution. Such difference can be further quantified by skewness and tendency, providing an alternative method for differential diagnosis.

## Methods

### [^18^F]BBPA PET/CT and MRI acquisition

[^18^F]BBPA PET/CT and MRI were performed within 1 week on separate days. For [^18^F]BBPA PET/CT, a dose of 3.7 MBq (0.1 mCi)/kg [^18^F]BBPA was intravenously given, and a PET/CT scan was acquired using a Biograph mCT Flow 64 scanner (Siemens, Germany) 30 min after injection. The PET image was transferred into an SUV map that was normalized by body weight and decay factor. For MRI, contrast-enhanced T1-weighted MRI (matrix 256 × 256, slice thickness 1 mm, gadolinium chelate 0.1 mmol/kg) and T2-weighted MRI (matrix 256 × 256, slice thickness 5–6 mm) were acquired from a 3.0 T Discovery MR750 scanner (GE, USA).

[^18^F]BBPA PET/CT image and T2-weighted MRI were co-registered to the thin-slice contrast-enhanced T1-weighted MRI to unify the origin and direction of images, allowing the same region of interest (ROI) refers to identical area in different image modality.

### Patients enrollment

Patients that were suspected to have primary or metastatic brain tumors were enrolled under the following criteria: (1) age ≥ 18 years; (2) Karnofsky Performance Score (KPS) ≥ 80; (3) suspected to have malignant gliomas or metastatic brain tumors based on medical history, clinical and radiological evaluation; (4) no contradictions for PET/CT and MRI scan. The pathological diagnosis was established by two neuropathologists according to the 2021 WHO classification for central nervous system tumors [24]. The therapeutic strategies, including but not limited to, surgery, radiotherapy, pharmacological treatment, or close imaging follow-up, were determined by a multi-disciplinary team after PET/CT and MRI scans.

### Tumor segmentation

Three spherical reference regions of interest (ROIref) with a diameter of 1 cm were manually placed on the contralateral area (mirroring the position of the tumor) to calculate the maximum and mean SUV of the normal brain (generating Nmax and Nmean, respectively) [22].

The ROI of the lesion was delineated by the definition of gross total resection (GTR) for brain tumors, which includes the contrast-enhanced region for significantly contrast-enhanced tumors or the region with abnormal T2-weighted signal for non-significantly contrast-enhanced tumors. The ROI was semi-automatically delineated and manually revised by a neurosurgeon on the thin-slice T1-weighted MRI using 3D Slicer (4.11.2, www.slicer.org). The ROI was subsequently applied to the co-registered BBPA PET images for feature calculation and histogram analysis.

### Traditional feature calculation

Five traditional quantitative parameters, namely SUVmax, SUVmean, metabolic tumor volume (MTV), total lesion activity (TLA) and tumor-to-normal brain ratio (T/N ratio), were calculated [25]. SUVmax and SUVmean represent the maximum and mean SUV of ROI, while MTV and TLA calculate the volume and total radioactivity inside ROI. The T/N ratio was calculated as the ratio of SUVmax and Nmax.

### Histogram plotting and quantification

The SUV of each voxel within ROI was documented as a number series, and a histogram was plotted to visualize the voxel value distribution. Skewness and tendency were defined to reflect the histogram characteristics:

$${\text{Skewness}}=\frac{\frac{1}{{{\text{N}}}_{{\text{p}}}}\sum_{{\text{i}}=1}^{{{\text{N}}}_{{\text{p}}}}({\text{X}}({\text{i}})-\overline{{\text{X}}}{)}^{3}}{{\left(\sqrt{\frac{1}{{{\text{N}}}_{{\text{p}}}}\sum_{{\text{i}}=1}^{{{\text{N}}}_{{\text{p}}}}({\text{X}}({\text{i}})-\overline{{\text{X}}}{)}^{2}}\right)}^{3}}$$

where X refers to all voxel values included in the ROI, ${{\text{N}}}_{{\text{p}}}$ refers to the number of voxel within ROI.

$${\text{Tendency}}={{\text{SUV}}}_{{\text{mean}}}-{{\text{SUV}}}_{{\text{median}}}$$

where SUVmean and SUVmedian refer to the mean and median SUV value within ROI.

### Statistical analysis

Images were processed and segmented on 3D slicer (4.11.2, www.slicer.org). The Wilcoxon rank-sum test was applied to evaluate whether a parameter was significantly different in distinct circumstances. Statistical analysis were performed using Python (3.8.5, www.python.org) and R (4.0.4, www.r-project.org).

## Results

### Elevated [^18^F]BBPA activity in both neoplastic and non-neoplastic lesions

Twenty-one patients who were suspected of primary or recurrent malignant brain tumors were enrolled. Ten patients were primary brain tumors (all pathologically confirmed), 8 patients were metastatic brain tumors (5 pathologically confirmed, 3 diagnosed according to patient history and imaging characteristics), and 3 patients were non-neoplastic lesions (1 pathological confirmed, 2 verified based on history, imaging behavior and treatment outcome). The baseline characteristics of the enrolled patients are displayed in Table 1.

**Table 1:** Baseline characteristics of the enrolled patients

Characteristics	Population
Age (mean ± SD)	54.8 ± 12.5
Sex	
Male	11 (52.4%)
Female	10 (47.6%)
Primary/recurrent diffuse gliomas	10 (47.6%)
WHO grade III	1 (4.8%)
IDH-mutant, 1p/19q-codeleted	1 (4.8%)
WHO grade IV	9 (42.9%)
IDH-wildtype	7 (33.3%)
IDH-mutant, 1p/19q-intact	1 (4.8%)
H3K27M-mutant	1 (4.8%)
Metastatic brain tumors	8 (38.1%)
Lung origin	2 (9.5%)
Breast origin	2 (9.5%)
Pancreatic origin	1 (4.8%)
Esophageal origin	1 (4.8%)
Renal origin	1 (4.8%)
Lymphatic origin	1 (4.8%)
Non-neoplastic lesion	3 (14.3%)
Radiation necrosis	2 (9.5%)
Viral encephalitis	1 (4.8%)

Unless otherwise noted, data in the table refers to the number and percentages of patients/tumors

*SD* standard deviation, *WHO* World Health Organization

All lesions exhibited elevated [^18^F]BBPA radioactivity, with SUVmax of 2.56 ± 0.57, T/N ratio of 19.7 ± 5.1 in the whole population. However, the traditional metabolic parameters (SUVmax, SUVmean, MTV, TLA and T/N ratio) were not able to distinguish neoplastic and non-neoplastic lesions (p = 0.269–0.975) SUVmax were 2.52 ± 0.61 and 2.75 ± 0.21, and T/N ratio were 19.2 ± 5.3 and 22.7 ± 2.2 in neoplastic and non-neoplastic lesions, respectively. Traditional [^18^F]BBPA metabolic parameters in neoplasms and non-neoplastic lesions are demonstrated in Table 2.

**Table 2:** Traditional metabolic parameters of [^18^F]BBPA in neoplasms and non-neoplastic lesions

Diagnosis	SUVmax	SUVmean	MTV	TLA	T/N ratio
Malignant brain tumor	2.52 ± 0.61	1.08 ± 0.31	29.6 ± 38.9	31.4 ± 35.6	19.2 ± 5.3
Non-neoplastic lesion	2.75 ± 0.21	0.89 ± 0.31	30.4 ± 50.9	17.3 ± 28.1	22.7 ± 2.2
P value	0.543	0.328	0.975	0.524	0.269

Statistical properties of each parameter were displayed as mean ± standard deviation. Independent sample t test was utilized to compare the differences between groups

*SUV* standard uptake value, *MTV* metabolic tumor volume, *TLA* total lesion activity, *T/N ratio* tumor-to-normal brain ratio

### [^18^F]BBPA histogram distinguishes neoplastic and non-neoplastic lesions

**Fig. 1:**
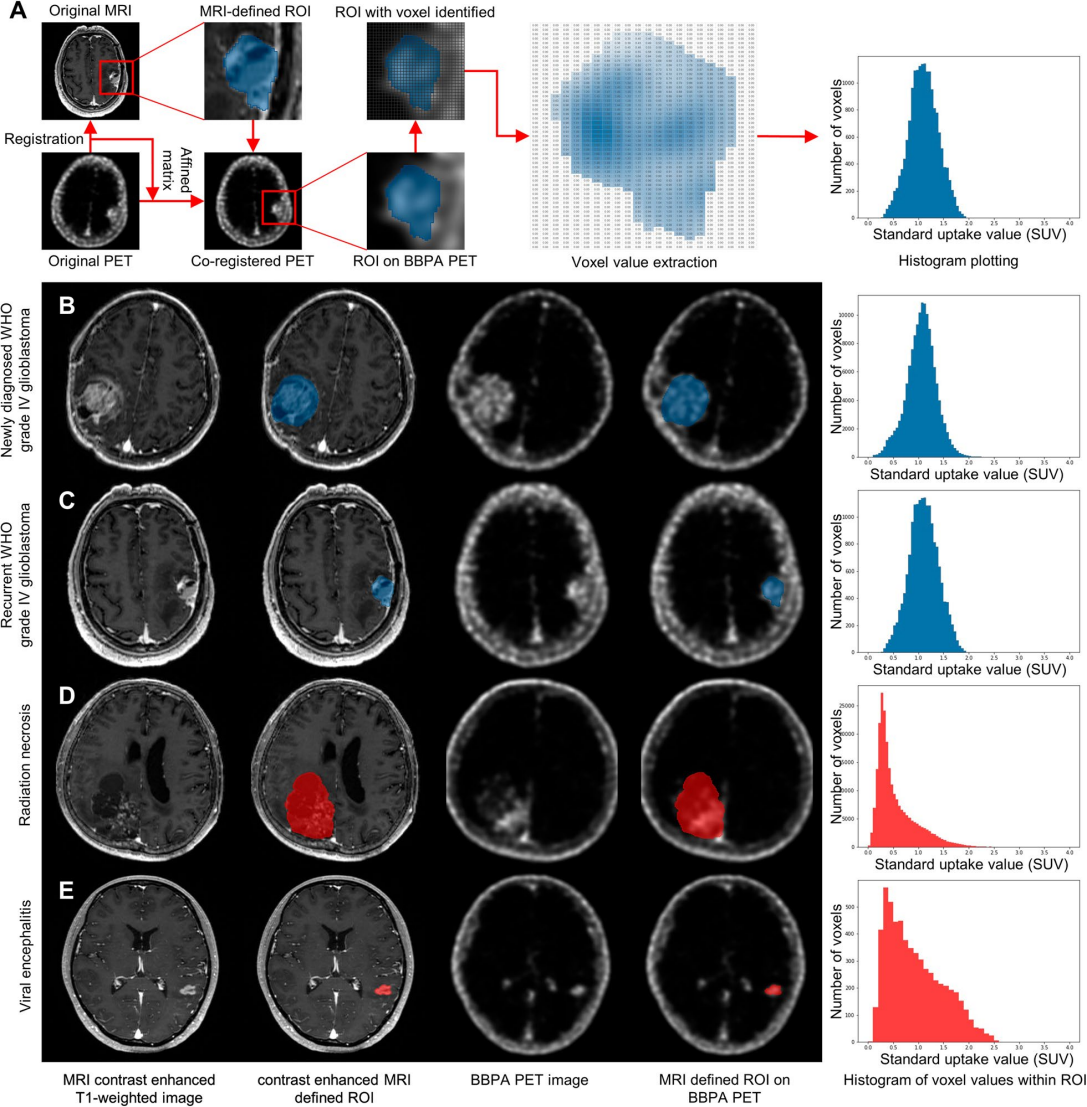
[^18^F]BBPA histogram distinguishes neoplastic lesions and non-neoplastic lesions. **A** Flowchart of histogram plotting demonstrated that the [^18^F]BBPA PET was first co-registered to contrast-enhanced T1-weighted thin slice MRI, and the region of interest (ROI) was defined by the gross total resection (GTR) area on MRI. The ROI was subsequently applied to [^18^F]BBPA PET, and the voxel value within the ROI was documented. A histogram of voxel values was plotted, which reflected the distribution of values within ROI (X-axis ranged 0–4, Y axis ranged according to the number of voxels). **B** A newly diagnosed glioblastoma (WHO grade IV, IDH wild-type) displayed significant MRI contrast enhancement, and the ROI was semi-automatically defined (blue area) and applied on [^18^F]BBPA PET. Histogram of voxels within ROI revealed a pattern similar to normal distribution. **C** Similarly, the ROI (blue area) in a recurrent glioblastoma (WHO grade IV, IDH wild-type) patient was defined and the histogram can also be fitted as a normal distribution. **D** On the other hand, a gross resected pathological confirmed radiation necrosis also displayed BBPA activity with SUVmax of 2.97, but the histogram from the ROI (red area) was positively skewed and the SUVmean was 0.56. **E** Similarly, a viral encephalitis whose lesion completely remission after anti-viral therapy was also contrast-enhanced and [^18^F]BBPA active, and the histogram of lesion (red area) was also positively skewed (SUVmax 2.55, SUVmean 0.94)

The histogram that reflects the voxel value distribution within ROI was plotted to visualize the metabolic characteristics of [^18^F]BBPA-PET. The neoplastic lesions (including both primary and metastatic tumors) exhibited a symmetrical distribution that can be fitted as a normal distribution. On the other hand, the non-neoplastic lesions (radiation necrosis and viral encephalitis) displayed a positive skewed (left deviated) distribution which was conspicuously varied from a normal distribution. Flowchart and examples of [^18^F]BBPA-PET histogram are displayed in Fig. 1.

Skewness represents the extent of the histogram varied from a normal distribution, with positively skewed (left deviated) and negatively skewed (right deviated) distributions exhibiting positive and negative values, respectively. The neoplastic histograms revealed higher similarity to a normal distribution with a skewness of 0.145 ± 0.337, while the non-neoplastic cases were significantly positively skewed with a skewness of 0.935 ± 0.448 (*P* = 0.002). Tendency, calculated as the subtraction of SUVmean and SUVmedian, exhibited a significantly smaller value in neoplastic lesions than non-neoplastic lesions (0.001 ± 0.038 vs. 0.123 ± 0.021, *P* < 0.001). Statistical properties of skewness and tendency are illustrated in Table 3.

**Table 3:** Histogram parameters of [^18^F]BBPA in neoplasms and non-neoplastic lesions

Diagnosis	Skewness	Tendency
Malignant brain tumor	0.145 ± 0.337	0.001 ± 0.038
Non-neoplastic lesion	0.935 ± 0.448	0.123 ± 0.022
P value	0.002	< 0.001
Threshold	0.624	0.084

Statistical properties of each parameter were displayed as mean ± standard deviation. Independent sample *t* test was utilized to compare the differences between groups

**Fig. 2:**
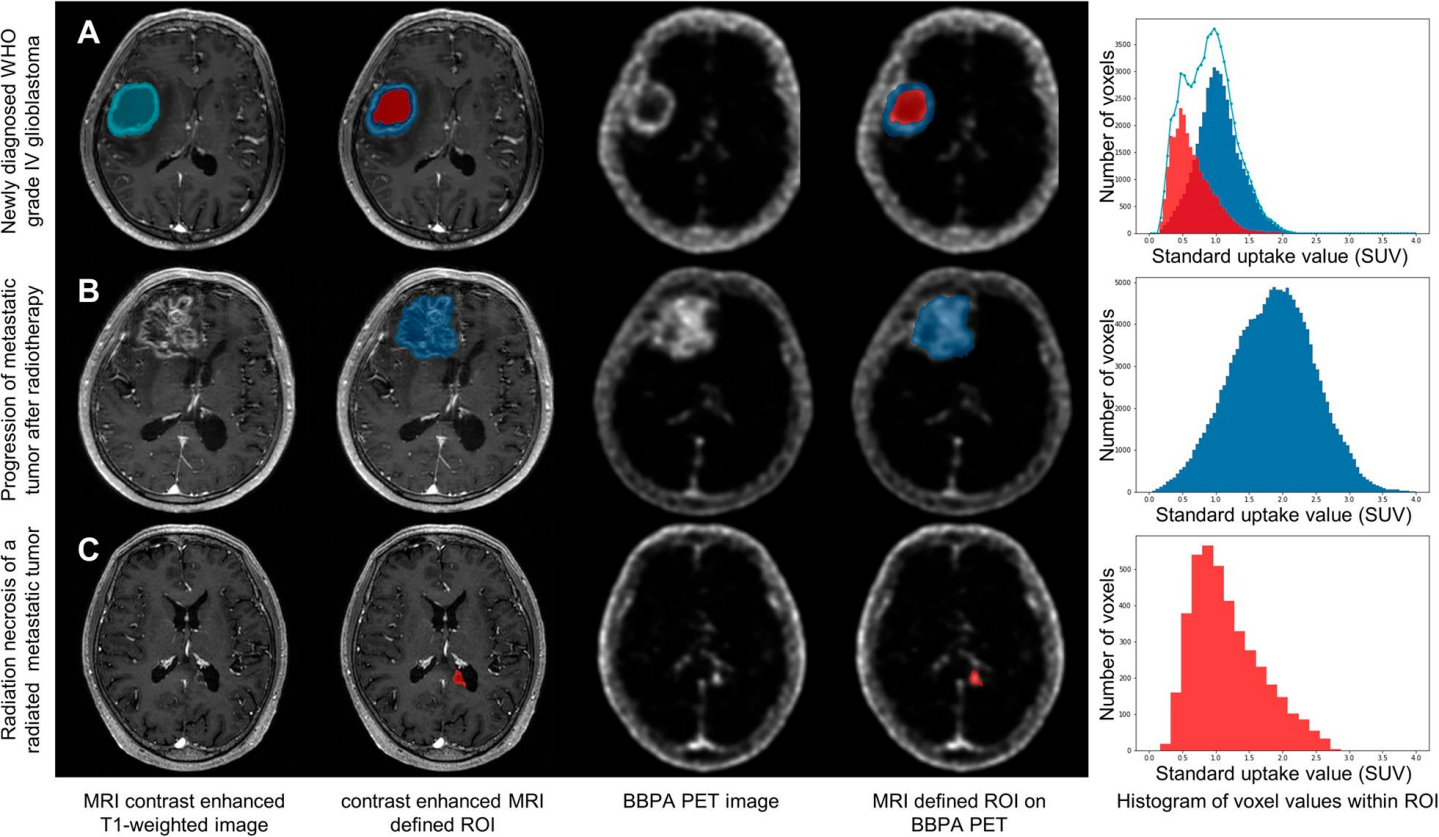
[^18^F]BBPA histogram for differential diagnosis in clinical scenario. **A** A 62/M patient displayed right frontal lesion with ring-like contrast enhanced on MRI, and the whole lesion (light blue area), contrast enhanced area (blue area) and non-contrast enhanced area (red area) were semi-automatically defined. The contrast enhanced (blue) area exhibited a BBPA uptake similar to normal distribution which is in accordance with tumor characteristics, while the central (red) region revealed a positive skewed [^18^F]BBPA activity that is corresponding to non-neoplastic lesion. The whole tumor displayed a dual-peaked histogram pattern (light blue line) that can be divided into two single peaks on the separate segmentations (red and blue area), and this metabolic characteristics was suggestive of glioblastoma. **B** A 71/F patient exhibited right frontal metastatic breast cancer and received cranial radiotherapy and tyrosine kinase inhibitor. Four months after treatment, the tumor was considered to have treatment response thanks to the slightly improved volume effect on MRI. However, the lesion displayed increased symmetric [^18^F]BBPA activity, suggesting there was remaining active tumor. The patient continued tyrosine kinase inhibitor treatment, and six months after [^18^F]BBPA PET, the patient progressed clinically and radiologically. **C** A 63/M patient with periventricular metastatic lung cancer received radiotherapy and achieved completed response on MRI. Fifteen months after radiotherapy, the patient developed regional abnormal signal on MRI that was initially considered as tumor recurrence. However, the lesion exhibited positive skewed [^18^F]BBPA distribution that was suggestive of non-neoplasms, and the lesion remained radiologically stable at 1-year follow-up (without anti-tumor treatment)

The capability of [^18^F]BBPA histogram to distinguish neoplastic and non-neoplastic lesions was further verified in 3 recent clinical scenarios. In a newly diagnosed glioblastoma (World Health Organization [WHO] grade IV, isocitrate dehydrogenase [IDH] wild-type), [^18^F]BBPA histogram separated the central necrosis (skewness 1.019, tendency 0.064) from the ring-like proliferating tumors (skewness 0.191, tendency 0.013), whom metabolic characteristics was suggestive of glioblastoma. In a post-radiation metastatic breast cancer, [^18^F]BBPA histogram identified tumor progression (skewness −0.043, tendency −0.017) earlier than MRI. In another post-radiation metastatic lung cancer, [^18^F]BBPA histogram recognized the lesion as radiation necrosis instead of tumor recurrence (skewness 0.721, tendency 0.109) and guide patient management (no anti-tumor treatment was given and the lesion remained radiologically stable at 1 year follow-up). Images and histograms of the 3 cases are displayed in Fig. 2.

## Discussion

Differentiating neoplastic and non-neoplastic lesions (i.e., inflammation, necrosis, anti-tumor immune response) remains a critical clinical issue at both initial diagnosis and treatment follow-up. Amino acid tracers such as [^18^F]FET were investigated to distinguish tumor progression and treatment-related changes, with a T/N ratio displayed accuracy of 0.70 and area under the ROC curve (AUC) of 0.75 at a cutoff value of 1.95 [26]. However, considerable situations were not identified by traditional parameters, and both neoplastic and non-neoplastic lesions exhibited elevated [^18^F]BBPA activity. Histogram was further proposed for differential diagnosis, and the SUV of a normal or neoplastic area with regional heterogeneity (e.g., [^18^F]FDG in the brain, [^18^F]FDG or [^18^F]FLT in head and neck squamous cell carcinoma) are expected to be normal distribution [27, 28]. The non-neoplastic lesions displayed positively skewed (left deviated) voxel value distribution that was visually differed from the normally distributed neoplastic lesions on the histogram, and can be further quantified by skewness and tendency, providing an alternative method for differential diagnosis. The clinical impact is further demonstrated in recent cases, in which [^18^F]BBPA PET identified the lesion properties earlier than traditional methods. Therefore, the histogram of [^18^F]BBPA PET might aid the differentiation of neoplastic and non-neoplastic lesions and ultimately facilitate the accurate treatment decisions.

The histogram analysis may be applied to other circumstances (i.e., other disease or radiotracers) with low background activity and high lesion uptake, and the segmentation is preferably conducted on alternative imaging modality rather than PET image (threshold-based PET segmentation would result in a clear boundary on histogram). However, the current study had several limitations including a small sample size (particularly for non-neoplastic lesions) and a short follow-up period (unable to demonstrate the prognostic value of [^18^F]BBPA histogram). For future works, a well-designed prospective study with balanced cohort and longitudinal follow-up is necessary to validate the findings, and an in-depth exploration of the mechanism underlying the [^18^F]BBPA histogram differences is necessitated. In conclusion, the histogram of [^18^F]BBPA PET can differentiate non-neoplastic lesions from proliferating tumors and would facilitate the precision diagnosis and patient management.

## Data Availability

The datasets generated during and/or analyzed during the current study are available from the corresponding author on reasonable request.

## Abbreviations

AUC: Area under the ROC curve
BAA: Boramino acids
BBPA: Trifluoroborate boronophenylalanine
BNCT: Boron neutron capture therapy
BPA: 4‑Boronophenylalanine
GTR: Gross total resection
IDH: Isocitrate dehydrogenase
KPS: Karnofsky Performance Score
MRI: Magnetic resonance imaging
MTV: Metabolic tumor volume
PET: Positron emission tomography
RANO: Response Assessment in Neuro-Oncology
RECIST: Response Evaluation Criteria in Solid Tumors
ROI: Region of interest
SUV: Standard uptake value
TLA: Total lesion activity
T/N ratio: Tumor-to-normal brain ratio
WHO: World Health Organization

## References

[1] Chiou VL, Burotto M (2015). Pseudoprogression and immune-related response in solid tumors. J Clin Oncol.

[2] Nishino M, Hatabu H, Johnson BE, McLoud TC (2014). State of the art: response assessment in lung cancer in the era of genomic medicine. Radiology.

[3] Brandsma D, Stalpers L, Taal W, Sminia P, van den Bent MJ (2008). Clinical features, mechanisms, and management of pseudoprogression in malignant gliomas. Lancet Oncol.

[4] Chen X, Lim-Fat MJ, Qin L (2021). A comparative retrospective study of immunotherapy RANO versus standard RANO criteria in glioblastoma patients receiving immune checkpoint inhibitor therapy. Front Oncol.

[5] Wen PY, van den Bent M, Youssef G (2023). RANO 2.0: update to the response assessment in neuro-oncology criteria for high- and low-grade gliomas in adults. J Clin Oncol.

[6] Youssef G, Rahman R, Bay C (2023). Evaluation of standard response assessment in neuro-oncology, modified response assessment in neuro-oncology, and immunotherapy response assessment in neuro-oncology in newly diagnosed and recurrent glioblastoma. J Clin Oncol.

[7] Nasseri M, Gahramanov S, Netto JP (2014). Evaluation of pseudoprogression in patients with glioblastoma multiforme using dynamic magnetic resonance imaging with ferumoxytol calls RANO criteria into question. Neuro Oncol.

[8] Yang S, Ma Y, Xu Y (2023). Dosimetric and clinical analysis of pseudo-progression versus recurrence after hypo-fractionated radiotherapy for brain metastases. Radiat Oncol.

[9] Seymour L, Bogaerts J, Perrone A (2017). iRECIST: guidelines for response criteria for use in trials testing immunotherapeutics. Lancet Oncol.

[10] Wen PY, Macdonald DR, Reardon DA (2010). Updated response assessment criteria for high-grade gliomas: response assessment in neuro-oncology working group. J Clin Oncol.

[11] Tensaouti F, Khalifa J, Lusque A (2017). Response Assessment in neuro-oncology criteria, contrast enhancement and perfusion MRI for assessing progression in glioblastoma. Neuroradiology.

[12] Rowe LS, Butman JA, Mackey M (2018). Differentiating pseudoprogression from true progression: analysis of radiographic, biologic, and clinical clues in GBM. J Neurooncol.

[13] Rodriguez D, Chambers T, Warmuth-Metz M (2019). Evaluation of the implementation of the response assessment in neuro-oncology criteria in the HERBY trial of pediatric patients with newly diagnosed high-grade gliomas. AJNR Am J Neuroradiol.

[14] Chawla S, Bukhari S, Afridi OM (2022). Metabolic and physiologic magnetic resonance imaging in distinguishing true progression from pseudoprogression in patients with glioblastoma. NMR Biomed.

[15] Liu Z, Chen H, Chen K (2015). Boramino acid as a marker for amino acid transporters. Sci Adv.

[16] Li J, Shi Y, Zhang Z (2019). A metabolically stable boron-derived tyrosine serves as a theranostic agent for positron emission tomography guided boron neutron capture therapy. Bioconjug Chem.

[17] Lan X, Fan K, Cai W (2021). First-in-human study of an (18)F-labeled boramino acid: a new class of PET tracers. Eur J Nucl Med Mol Imaging.

[18] Liu Z, Ehlerding EB, Cai W, Lan X (2018). One-step synthesis of an (18)F-labeled boron-derived methionine analog: a substitute for (11)C-methionine?. Eur J Nucl Med Mol Imaging.

[19] Chen J, Li C, Hong H (2019). Side chain optimization remarkably enhances the in vivo stability of (18)F-labeled glutamine for tumor imaging. Mol Pharm.

[20] Chen M, Wang C, Wang X, Tu Z, Ding Z, Liu Z. An "AND" logic-gated prodrug micelle locally stimulates anti-tumor immunity. *Adv Mater*. 2023:e2307818. 10.1002/adma.202307818 37935201

[21] Li Z, Kong Z, Chen J (2021). (18)F-boramino acid PET/CT in healthy volunteers and glioma patients. Eur J Nucl Med Mol Imaging.

[22] Kong Z, Li Z, Chen J (2022). Metabolic characteristics of [(18)F]fluoroboronotyrosine (FBY) PET in malignant brain tumors. Nucl Med Biol.

[23] Li Z, Chen J, Kong Z (2024). A bis-boron boramino acid PET tracer for brain tumor diagnosis. Eur J Nucl Med Mol Imaging.

[24] Louis DN, Perry A, Wesseling P (2021). The 2021 WHO classification of tumors of the central nervous system: a summary. Neuro Oncol.

[25] Kong Z, Zhang Y, Liu D (2021). Role of traditional CHO PET parameters in distinguishing IDH, TERT and MGMT alterations in primary diffuse gliomas. Ann Nucl Med.

[26] Maurer GD, Brucker DP, Stoffels G (2020). (18)F-FET PET imaging in differentiating glioma progression from treatment-related changes: a single-center experience. J Nucl Med.

[27] Scarpelli M, Eickhoff J, Cuna E, Perlman S, Jeraj R (2018). Optimal transformations leading to normal distributions of positron emission tomography standardized uptake values. Phys Med Biol.

[28] Proesmans S, Raedt R, Germonpré C (2021). Voxel-Based Analysis of [18F]-FDG brain PET in rats using data-driven normalization. Front Med (Lausanne).

